# Minimizing sperm oxidative stress using nanotechnology for breeding programs in rams

**DOI:** 10.1186/s40104-023-00907-3

**Published:** 2023-08-10

**Authors:** Alejandro Jurado-Campos, Pedro Javier Soria-Meneses, María Arenas-Moreira, Carlos Alonso-Moreno, Virginia Rodríguez-Robledo, Ana Josefa Soler, José Julián Garde, María del Rocío Fernández-Santos

**Affiliations:** 1 SaBio IREC (CSIC—UCLM—JCCM), Campus Universitario, S/N, 02071 Albacete, Spain; 2 Departamento de Química Inorgánica, Orgánica Y Bioquímica-Centro de Innovación en Química Avanzada (ORFEO-CINQA), Facultad de Farmacia, Universidad de Castilla-La Mancha, 02008 Albacete, Spain; 3 Centro Regional de Investigación Biomédicas, Unidad nanoDrug, Universidad de Castilla-La Mancha, 02008 Albacete, Spain; 4 Facultad de Farmacia, Universidad de Castilla La Mancha, 02071 Albacete, Spain

**Keywords:** Breeding technology, Nanoemulsions, Nanotechnology, Sperm oxidative stress, Vitamin E

## Abstract

**Background:**

Artificial insemination (AI) is a routine breeding technology in animal reproduction. Nevertheless, the temperature-sensitive nature and short fertile lifespan of ram sperm samples hamper its use in AI. In this sense, nanotechnology is an interesting tool to improve sperm protection due to the development of nanomaterials for AI, which could be used as delivery vehicles. In this work, we explored the feasibility of vitamin E nanoemulsion (NE) for improving sperm quality during transport.

**Results:**

With the aim of evaluating this proposal, ejaculates of 7 mature rams of Manchega breed were collected by artificial vagina and extended to 60 × 10^6^ spz/mL in Andromed®. Samples containing control and NE (12 mmol/L) with and without exogenous oxidative stress (100 µmol/L Fe^2+^/ascorbate) were stored at 22 and 15 ºC and motility (CASA), viability (YO-PRO/PI), acrosomal integrity (PNA-FITC/PI), mitochondrial membrane potential (Mitotracker Deep Red 633), lipoperoxidation (C_11_ BODIPY 581/591), intracellular reactive oxygen species (ROS) production and DNA status (SCSA®) monitored during 96 h. Our results show that NE could be used to maintain ram spermatozoa during transport at 15 and 22 ºC for up to 96 h, with no appreciable loss of kinematic and physiological characteristics of freshly collected samples.

**Conclusions:**

The storage of ram spermatozoa in liquid form for 2–5 d with vitamin E nanoemulsions may lead more flexibility to breeders in AI programs. In view of the potential and high versatility of these nanodevices, further studies are being carried out to assess the proposed sperm preservation medium on fertility after artificial insemination.

**Graphical Abstract:**

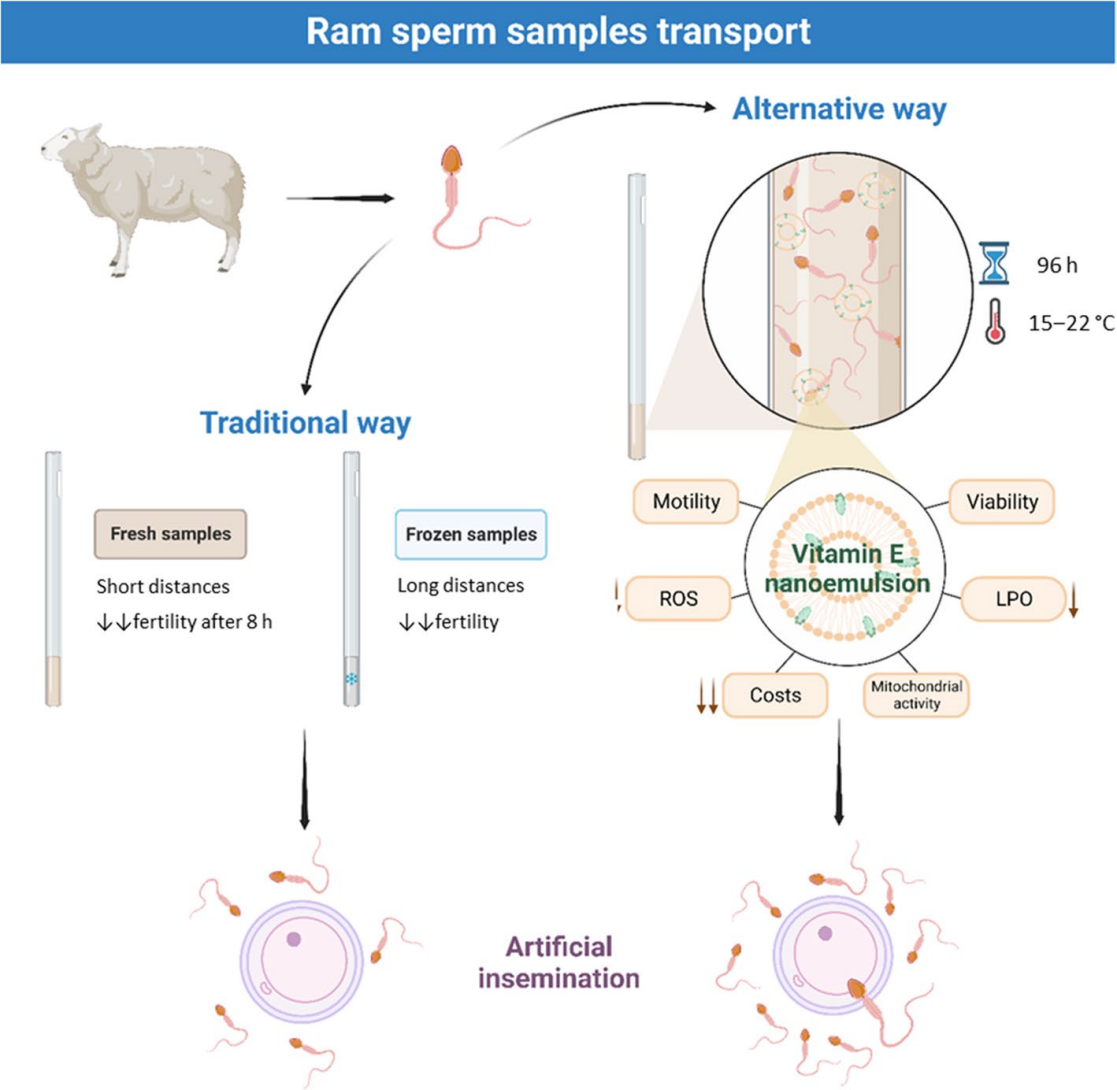

## Introduction

Artificial insemination (AI) determines the success of a breeding program by far. AI is a technique developed to facilitate the dissemination of genetic improvement, as well as to allow the connection of the population necessary for the correct genetic evaluation of breeding stock. AI requires an appropriate management model and animals with high productive performance for the traits of interest [1].

Despite the great advantages achieved in AI, many methods face several limitations for a clear translation to breeding programs. AI constrains depend on multiple factors, such as the compulsory use of synchronization treatments, semen quality and conservation, adaptation to the functional and physiological characteristics of the native breeds linked to special production systems. However, low fertility rates and the high variability found between batches in AI is mainly attributed to collection, processing, conservation and application of semen doses [2]. In this regard, monitoring conservation would facilitate transporting longer distances to lead to successful inseminations [3].

Nowadays, the two most widely alternatives used to transport semen samples are: for shorter distances, samples are refrigerated between 0–15 °C, slowing down biochemical functions of spermatozoa which implies a reduction for fertility of 10%–35% per day [1]; for longer distances, cryopreservation is the most extended alternative being the samples frozen on straws at −196 °C by using liquid nitrogen [4]. In both scenarios, when spermatozoa are cooled, the total amount of immobile cells and abnormally shaped is increased. In addition, the distribution of membrane lipids is altered and the levels of intracellular calcium increases. These changes are currently known as cold shock [5].

Regrettably, ram spermatozoa are more sensitive to cold shock than other mammals species due to a very low cholesterol/phospholipid ratio and a low content of saturated fatty acid in the membrane [6]. On ram spermatozoa, cold shock can diminish sperm motility and viability up to 60% [7]. Besides, it can increase the risk of DNA fragmentation with all its consequences for the spermatozoon [8, 9]. These characteristics cause relative short fertile lifespan of ram spermatozoa, which is a problem for industrial-scale sheep breading operations. Besides, mammalian spermatozoa are particularly sensitive to oxidative stress which result in collateral damage to sperm plasma membrane proteins and lipids [10]. It is a proven fact that during storage and transport the impact of oxidative stress on the sperm cell increases [11].

Minimizing oxidative stress and cold shock on storage to maintain ram sperm fertility would pave the way for ram AI. In this context, nanotechnology is a tool to be explored. During the last years, our research group have developed different vitamin E nanodevices for AI, such as nanoemulsions [12, 13] and hydrogels [12]. The viability and feasibility of such nanodevices were tested on red deer (*Cervus elaphus hispanicus*) and ram (*Ovis aries*), using both fresh and thawed samples. The use of these nanodevices showed beneficial effects on sperm motility parameters compared to free vitamin E while ROS production and lipid peroxidation were reduced under oxidative stress conditions. Vitamin E is renowned for its exceptional antioxidative properties, making it a gold standard in spermatology for its ability to safeguard sperm samples by effectively reducing ROS production and lipid peroxidation. Furthermore, studies have shown that vitamin E can significantly reduce DNA and acrosome damage, further highlighting its crucial role [14]. However, despite its remarkable benefits, this potent antioxidant is not without limitations. It is vulnerable to instability when exposed to oxidative processes or changes in pH, and can potentially exhibit prooxidant effects even at low concentrations. Additionally, its low solubility presents challenges, requiring administration with organic solvents such as ethanol or dimethyl sulfoxide, which have been shown to be toxic to sperm cells [15] and associated with decreased sperm motility [14, 16–19]. These problems with free vitamin E have been overcome using nanoemulsions. Herein, we made progress in the assessment on vitamin E nanoemulsion (NE) for AI. To do so, we studied its availability for storing with the purpose of assisting sperm transport. The transport of sperm samples is challenging and those were chosen as a proof of concept in this work. Therefore, the efficacy of the use of NE on the main structural and kinematic parameters of ram spermatozoa during a long-term incubation period (up to 96 h) is reported.

Although under normal conditions ovine sperm samples are not usually subjected to high levels of oxidative stress, our experiment aims to subject them to extreme oxidative stress to mimic the worst scenarios that sperm cells may face in vivo. These scenarios may include exposure to toxins, prolonged sample transport, suboptimal sample storage, poor nutrition, or presence of infectious diseases [20]. Moreover, it is known that males of high economic or genetic value may be more susceptible to oxidative stress due to their high reproductive demand. Therefore, it is important to evaluate the performance of vitamin E nanoemulsions under these conditions, as it would allow the development of an antioxidant protection system capable of acting in the worst possible scenarios. This would represent a significant breakthrough in the application of nanotechnology in the reproductive industry, particularly in the area of antioxidant protection for sperm.

## Materials and methods

### Reagents and media

Cytometry equipment (software and consumables) were acquired from Beckman Coulter (Fullerton, CA, USA), while Andromed® was purchased from (Minitüb, Tiefenbach, Germany). Acetone, FeSO_4_·7H_2_O, propidium iodide, *L*-α-phosphatidylcholine from soy bean, Pluronic® F-127 and vitamin E (CAS number 10191–41-0) were obtained from the Sigma Chemical Co. (Madrid, Spain). Fluorescent probes were purchases from Thermo Fisher Scientific (Barcelona, Spain), unless otherwise stated and their stock solutions are the follow: PI: 1.5 × 10^–3^ mol/L in Milli-Q water; YO-PRO-1: 5 × 10^–5^ mol/L in DMSO; PNA-FITC: 2 × 10^–7^ mol/L in DMSO; Mitotracker Deep Red 633: 1 × 10^–3^ mol/L in DMSO; C_11_ BODIPY 581/591: 2 × 10^–4^ mol/L in DMSO; CMH2DFCDA: 5 × 10^–4^ mol/L in DMSO. All the fluorescent stocks were kept at −20 °C, in the dark until needed.

The oxidant solution was prepared as 0.01 mol/L FeSO_4_ and 0.05 mol/L sodium ascorbate (Fe^2+^) in water. The TNE *buffer* was composed of 0.01 mol/L Tris–HCl, 0.15 mol/L NaCl and 1 × 10^–3^ mol/L EDTA (pH 7.5) [21]. Z-sizer NanoZS from Malvern (UK) were used for the characterization of the nanoemulsions.

### Vitamin E nanoemulsion formulation

The protocol described by Sanchez-Rubio et al. [13] was used to obtain the vitamin E nanoemulsions. Briefly, nanoemulsion were obtained by employing controlled emulsification, when an organic phase comprised ethanol/acetone (1:20), 93 mg of vitamin E and *L*-α-phosphatidylcholine (20 mg) were poured onto 10 mL of a 0.2% (w/v) aqueous solution of Poloxamer 127. Then, the mixture was homogenized with an Ultra-Turrax disperser for 10 min at 14,000 r/min and the organic solvents removed using a rotary evaporator for 30 min at 14,000 r/min. Parameters such as hydrodynamic radio (R_H_), polydispersity index (PDI), and Z-potential were obtained using dynamic light scattering (DLS) measurements.

### Animals

Animal handling was performed in accordance with Spanish Animal Protection Regulation, RD 53/2013, which conforms to European Union Regulation 2010/63. A total of seven Manchega rams (> 3 years of age) housed at the Experimental Farm of the University of Castilla-La Mancha (UCLM), were used in this study. All animal experimentation was performed following Ethics Committee University of Castilla‐La Mancha.

### Semen collection and initial evaluation

For this study, one ejaculate was used for male, which was collected by using an artificial vagina. After collection, sperm samples were transported to the laboratory at room temperature. Immediately, volume (mL), mass and individual sperm motility (scale 1–5), percentage of motile sperm (%) and sperm concentration (spz/mL) were evaluated [22], using a bright field and phase contrast microscopy (Eclipse 50i Nikon; Tokyo, Japan). Sperm concentration was calculated using a Makler counting chamber and the Sperm Class Analyzer software (SCA®) (Microptic, Barcelona, Spain). The samples with a minimum of 70% motile sperm and wave motion of 3.5, measured subjectively, were used in this study. Samples were not pooled for this study.

### Experimental design

This experiment was designed to explore the protective effect of vitamin E nanoemulsions during sperm incubation up to 96 h (22 ºC vs. 15 ºC) on sperm parameters. Sperm samples were diluted in Andromed® to 60 × 10^6^ spermatozoa/mL. The sperm solution was split among aliquots in microtubes. Two of them was left untreated as the control and subsequently incubate one of them at 22 ºC and the other at 15 ºC. The other two were diluted supplemented with nanoemulsions to a final concentration of vitamin E of 12 mmol/L (NE12), and then incubated to 22 or 15 ºC, respectively. All treatments were split in turn into two aliquots. One of them was subjected to oxidative stress by adding 100 µmol/L Fe^2+^ and 500 μmol/L ascorbate. Fe^2+^ oxidizes to Fe^3+^, which is recycled by the ascorbate, producing the highly reactive hydroxyl radical (HO). The microtubes were incubated, 22 or 15 ºC, and analysed after 0, 24, 48, 72 and 96 h. This experiment was replicated 7 times with samples from seven different males.

### Sperm motility analysis

The semen samples were diluted down to 15 × 10^6^ spz/mL in incubation media and loaded into a Makler counting chamber (10 µm depth) at 37 °C. Samples were captured and analysed using the Sperm Class Analyzer® (SCA V6.2, Microptic S.L; Barcelona, Spain). The image sequences were saved and later analysed and the software settings were adjusted for assessing ram sperm. The parameter used were: 25 frames/s; 30–100 µm^2^ for head area; VCL > 10 µm/s to classify a spermatozoon as motile. For each sperm, the software rendered the follows parameters: percentage of motile sperm, percentage of progressive motile sperm (PM), curvilinear velocity (VCL; µm/s), straight-line velocity path (VSL; µm/s) and average path velocity (VAP; µm/s) [23].

### Flow cytometry analysis

Two flow cytometers were employed in our study. Sperm viability and apoptosis-like changes, acrosome integrity, mitochondrial activity, lipid peroxidation and ROS production tests were carried out using a Cytoflex LX (Beckman Coulter, Inc., Brea, CA, USA), which was controlled with the CytExpert version 2.3.0.84 (Beckman Coulter). SCSA® was performed on a FC‐500 (Beckman Coulter) controlled with the MXP software (v.3). Raw data were analysed with IDEAS® software and WEASEL software (WEHI, Melbourne, Australia), respectively. In all cases, 10,000 events were acquired per sample. Mitotracker Deep Red was excited with a 633-nm helium–neon laser, Hoechst 33342 was excited with a 405-nm violet laser and all other fluorochromes were excited with a 488-nm laser. To exclude debris from sperm population, dot plots with forward-scatter light and side-scatter light or aspect ratio and area were employed in the respective cytometers.

### Fluorescence probes

#### Sperm viability and apoptosis-like changes

YO-PRO-1 and PI were used on sperm viability and apoptotic-like status tests. Spermatozoa were analysed by means of flow cytometry after 15 min of dark incubation period [24]. Specifically, we evaluated the fluorescent DNA markers for cells with compromised plasma membrane. Three subpopulations were obtained: viable (unstained: YO-PRO-1−⁄PI−), apoptotic-like membrane changes (YO-PRO-1+ ⁄ PI−) and non-viable (membrane damaged: PI+) [25].

#### Assessment of acrosome integrity

PI and PNA-FITC were used to determine the acrosome integrity. PNA (peanut agluttinin) binds specifically to the internal side of the external membrane of the acrosome, labelling acrosome-damaged spermatozoa. This fluorescent technique allows distinguishing among four sperm populations: alive with intact acrosomes (PNA−/PI−), dead with intact acrosomes (PNA−/PI+), alive with damaged acrosomes (PNA+ /PI−) and dead with damaged acrosomes (PNA+ /PI+) [26].

#### Assessment of mitochondrial activity

The mitochondrial status, investigated through the assessment of mitochondrial membrane potential (MMP), was evaluated using a Mitotracker Deep Red 633 fluorescent probe. The percentage of sperm Mitotracker+/YO-PRO-1− represented the proportion of viable sperm with active mitochondria [27]. The stained samples were incubated for 30 min in the dark before being run through the flow cytometer.

#### Assessment of lipid peroxidation

Lipid peroxidation was estimated using the C11-BODIPY 581/591 fluorescent probe. An aliquot of each sample was incubated for 30 min with C11-BODIPY 581/591. The sperm samples were washed by means of centrifugation (600 × *g*, 5 min) and were extended in BGM [14]. These steps were repeated at 4, 8 and 24 h and analyzed by using flow cytometry.

#### Production of reactive oxygen species

Reactive oxygen species production was recorded using the fluorescent probe 5-(and-6)-chloromethyl-2',7'-dichlorodihydrofluorescein acetyl ester (CM-H_2_DCFDA). CM-H_2_DCFDA penetrates the plasma membrane and is retained after intracellular esterases cleave the acetate groups and emits green fluorescent (504 nm) upon oxidation [14]. The intensity of fluorescence of CM-H_2_DCFDA increases simultaneously as ROS production. The samples were analyzed and the median H_2_DCFDA fluorescence of the viable sperm population value was noted [25].

#### Sperm chromatin structure assay

Chromatin stability was assessed following the SCSA*®* (Sperm Chromatin Structure Assay), based on the susceptibility of sperm DNA to acid-induced denaturation in situ and on the subsequent staining with the metachromatic fluorescent dye acridine orange [28].

Two parameters were analysed: a) DNA Fragmentation Index ( % of spermatozoa with DFI > 25) and HDS (High DNA Stainability: % of spermatozoa with green fluorescence higher than channel 600, of 1024 channels)[29].

### Statistical analysis

The assumption of normality and the equality of variance of data were checked prior to statistical analysis using the Kolmogorov–Smirnov normality test and a Levene's test, respectively. Later, vitamin E effect; time; oxidant; temperature; motility; viability; acrosome integrity; mitochondrial membrane potential; ROS and lipid peroxidation production; and DNA damage were analyzed by factorial ANOVA with IBM SPSS Statistics 25 (IBM, Armonk, NY, USA). In the model we included the random effect of male. When a significant effect was observed, post hoc Tukeys’ correction were carried out to adjust the *P*-values and account for multiple testing. Results are presented as mean ± SEM, and statistical significance was accepted for *P* < 0.05.

## Results

In this work, we explored the feasibility of NE for improving sperm quality during transport, which is a solution for increasing the life spam of ram spermatozoa.

### Effects of vitamin E nanoemulsions on ram sperm motility assessed by CASA

**Fig. 1:**
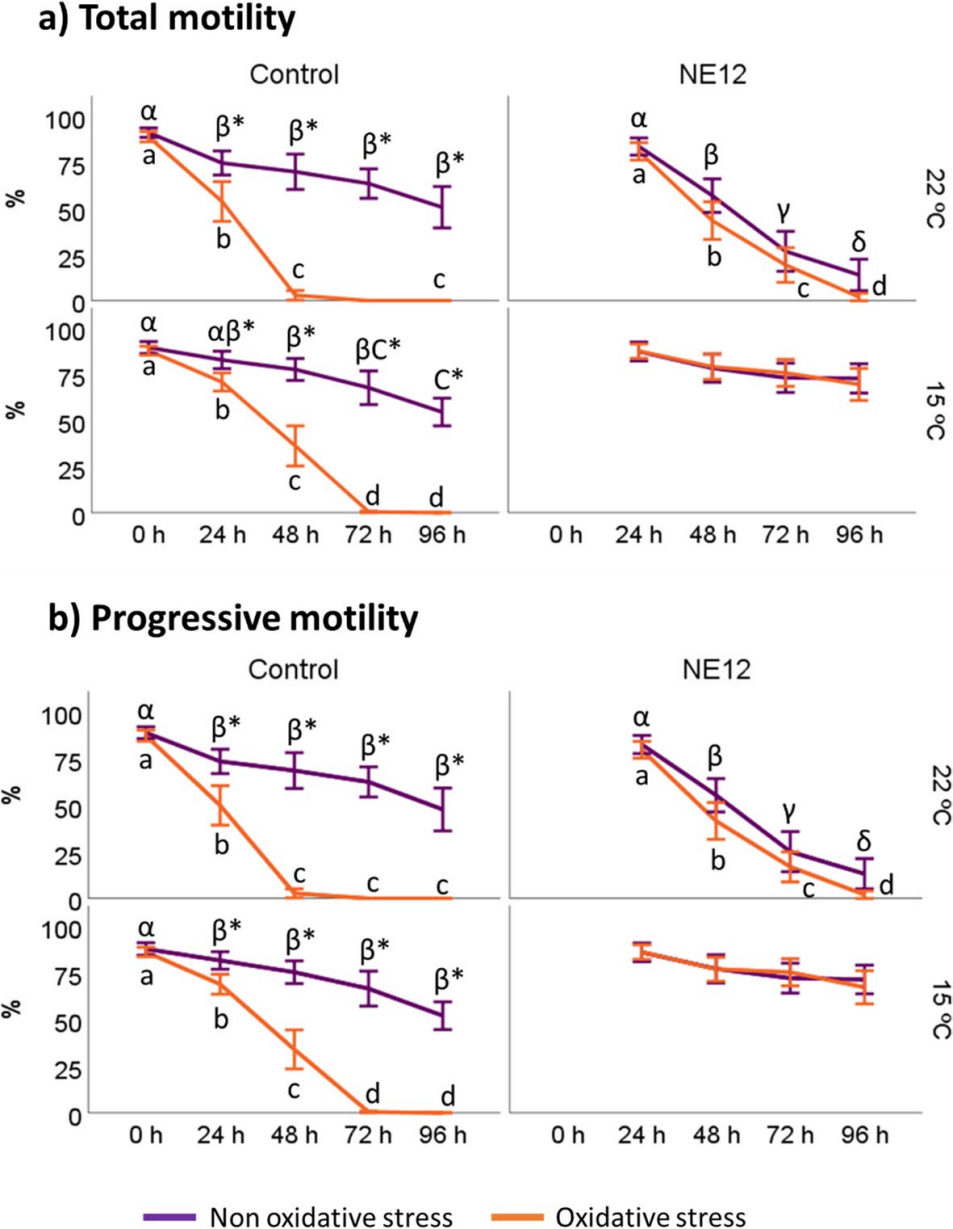
Effect of vitamin E nanoemulsion (NE) on kinematic parameters: **a**) total and **b**) progressive motility. Plots show the quadruple interaction of treatments × oxidative treatment × temperature × incubation time for CASA-derived variables. ^α,β,γ,δ^ (*P* < 0.05) indicate significant differences among hours of incubation within each treatment and temperature of incubation without oxidative stress (purple line). ^a–d^ (*P* < 0.05) indicates significant differences among hours of incubation within each treatment and temperature of incubation under oxidative stress (orange line). ^*^(*P* < 0.05) indicate differences between oxidative stress and non-oxidative stress treatments within each temperature and time of incubation

**Fig. 2:**
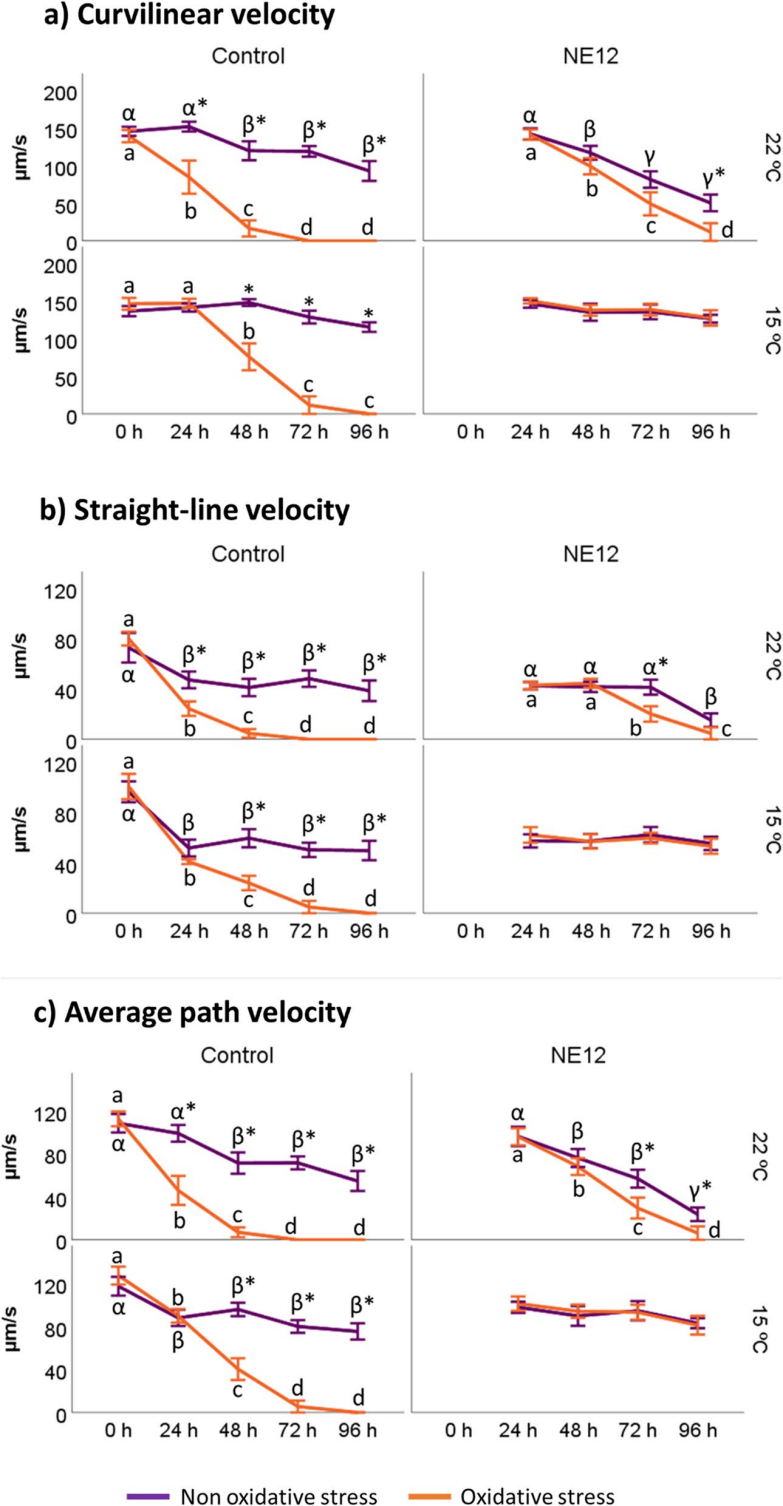
Effect of vitamin E nanoemulsion (NE) on kinematic parameters: **a**) curvilinear velocity (VCL), **b**) straight-line velocity (VSL) and **c**) average path velocity (VAP). Plots show the quadruple interaction of treatments × oxidative stress × incubation temperature × incubation time for kinematic sperm motility parameters. ^α,β,γ^ (*P* < 0.05) indicate significant differences among hours of incubation within each treatment and temperature of incubation without oxidative stress purple line). ^a–d^ (*P* < 0.05) indicates significant differences among hours of incubation within each treatment and temperature of incubation under oxidative stress (orange line). ^*^(*P* < 0.05) indicate differences between oxidative stress and non-oxidative stress treatments within each temperature and time of incubation

Figures 1 and 2 illustrate the impact of NE on the kinematic parameters of ram sperm, specifically motility and velocity. In the absence of exogenous oxidative stress, two distinct scenarios were observed (Fig. 1): a) when samples were incubated at 22 ºC, the motility parameters (total and progressive) exhibited a significantly higher percentage of motility in the control samples after 48 h; b) however, when samples were incubated at 15 ºC, these differences vanished and NE12 even displayed superior results compared to the control (72.78 ± 6.34 vs. 54.55 ± 9.83) after 96 h (*P* < 0.01).

Speed parameters VCL, VSL, and VAP showed no significant differences compared to the patterns observed for total and progressive motility (Fig. 2). In the absence of oxidative stress, NE12 samples did not yield improved results compared to the control samples. However, under oxidative stress conditions, NE12 demonstrated improvement over the control. For instance, as a representative example, after 96 h of incubation, the VCL in samples treated with NE12 (127.40 ± 27.10) was comparable to the results obtained by the control samples after 24 h (146.94 ± 15.72) and significantly better than the control after 72 h (11.67 ± 30.88).

### Effects of vitamin E nanoemulsion on ram sperm viability and acrosome integrity

**Fig. 3:**
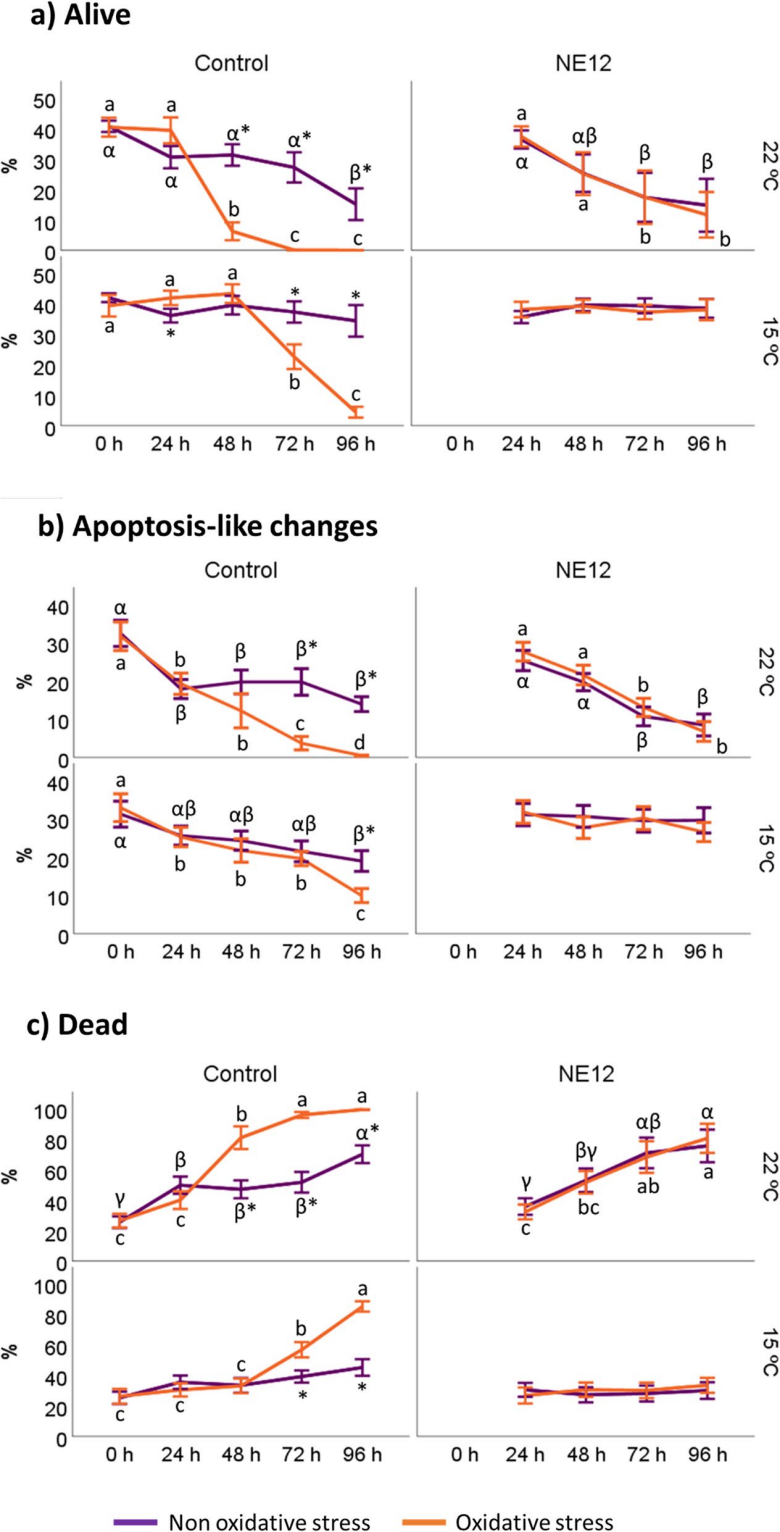
Effect of vitamin E nanoemulsion (NE) on sperm viability: **a**) % of alive spermatozoa, **b**) % of apoptosis-like changes and **c**) % of death spermatozoa. Plots show the quadruple interaction of treatments × oxidative stress × incubation temperature × incubation time for the flow cytometry analysis of sperm viability. ^α,β,γ^ (*P* < 0.05) indicate significant differences among hours of incubation within each treatment and temperature of incubation without oxidative stress (purple line). ^a–c^ (*P* < 0.05) indicates significant differences among hours of incubation within each treatment and temperature of incubation under oxidative stress (orange line). ^*^(*P* < 0.05) indicate differences between oxidative stress and non-oxidative stress treatments within each temperature and time of incubation

**Fig. 4:**
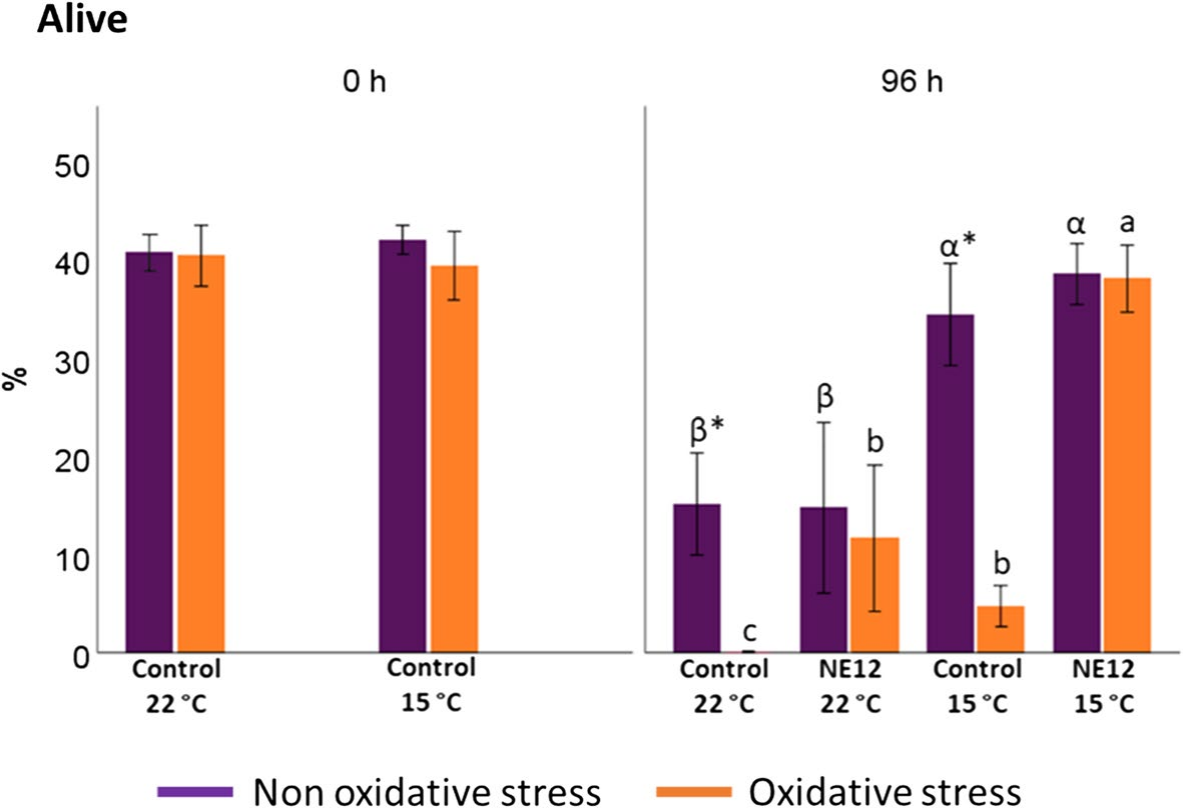
Effect of vitamin E nanoemulsion (NE) on sperm viability: comparison between control and NE12 over time. Plots show the quadruple interaction of treatments × oxidative stress × incubation temperature × incubation time for the flow cytometry analysis of sperm viability. ^α,β^ (*P* < 0.05) indicate significant differences among treatments × temperature without oxidative stress at the same incubation time (purple line). ^a–c^ (*P* < 0.05) indicates significant differences among treatments × temperature under oxidative stress at the same incubation time (orange line). ^*^(*P* < 0.05) indicate differences between oxidative stress and non-oxidative stress treatments within each treatment

Sperm viability was assessed using YO-PRO-1/IP staining (Fig. 3). After 96 h of incubation, the samples exhibited the following pattern: NE12 at 15 ºC ≈ control at 15 ºC > NE12 at 22 ºC ≈ control at 15 ºC. Control samples subjected to oxidative stress conditions showed a significant reduction in the percentage of viable cells after 24 h at 22 ºC and 48 h at 15 ºC, compared to non-oxidative stress samples. These differences were more pronounced when comparing to NE12 treatments. At 22 ºC, NE12 was able to maintain the viable cell population at levels comparable to non-oxidative conditions, while at 15 ºC, the viability after 96 h was similar to that observed at time 0 (control at 15 ºC 0 h: 39.31 ± 9.35 vs. NE12 at 15 ºC 96 h: 37.99 ± 9.03). Without oxidative stress, the samples displayed better viability results after 96 h at 15 ºC (*P* < 0.001). However, no significant differences were observed between the treatments (control vs. NE12) within the same temperature interval (Fig. 4).

**Fig. 5:**
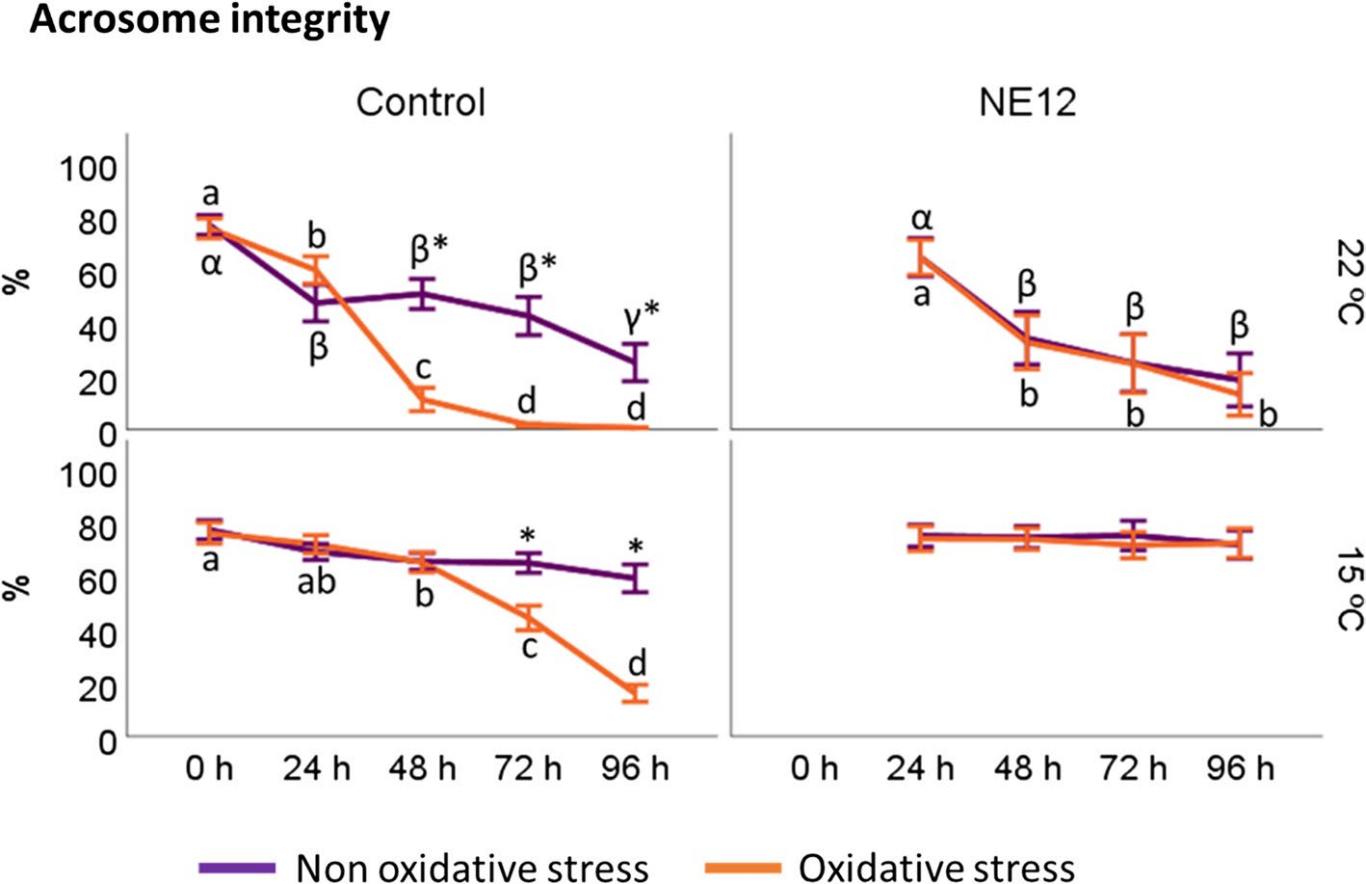
Effect of NE on acrosome integrity. Plots show the quadruple interaction of treatments × oxidative stress × incubation temperature × incubation time for the flow cytometry analysis of acrosome integrity (PNA− /PI−). ^α,β,γ^ (*P* < 0.05) indicate significant differences among hours of incubation within each treatment and temperature of incubation without oxidative stress (purple line). ^a–d^ (*P* < 0.05) indicates significant differences among hours of incubation within each treatment and temperature of incubation under oxidative stress (orange line). ^*^(*P* < 0.05) indicate differences between oxidative stress and non-oxidative stress treatments within each temperature and time of incubation

The acrosomal status exhibited a similar pattern to the viability results described earlier (Fig. 5). The percentage of spermatozoa with intact acrosomes was influenced by time and temperature, following the following pattern: NE12 at 15 ºC > control at 15 ºC > NE12 at 22 ºC > control at 15 ºC. In the absence of oxidative stress, no significant differences were observed between the treatments within the same temperature interval. The most notable finding regarding acrosomal status occurred under oxidative stress conditions at 15 ºC. In this scenario, the percentage of spermatozoa with intact acrosomes in the control samples decreased from 76.13 ± 10.47 to 16.17 ± 8.47 after 96 h, while NE12 treatments were able to maintain it at levels close to those observed at 0 h of incubation (NE12 at 15 ºC 96 h: 72.39 ± 14.45) (*P* < 0.001).

### Effects of vitamin E nanoemulsion on ram sperm mitochondrial activity

**Fig. 6:**
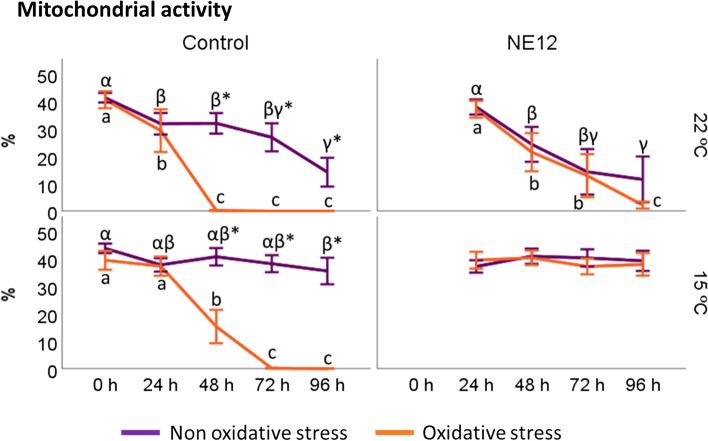
Effect of NE on mitochondrial activity. Plots show the quadruple interaction of treatments × oxidative stress × incubation temperature × incubation time for the flow cytometry analysis of mitochondrial activity (YO-PRO-1− /Mitotracker deep red+). ^α,β,γ^ (*P* < 0.05) indicate significant differences among hours of incubation within each treatment and temperature of incubation without oxidative stress (purple line). ^a–d^ (*P* < 0.05) indicates significant differences among hours of incubation within each treatment and temperature of incubation under oxidative stress (orange line). ^*^(*P* < 0.05) indicate differences between oxidative stress and non-oxidative stress treatments within each temperature and time of incubation

**Fig. 7:**
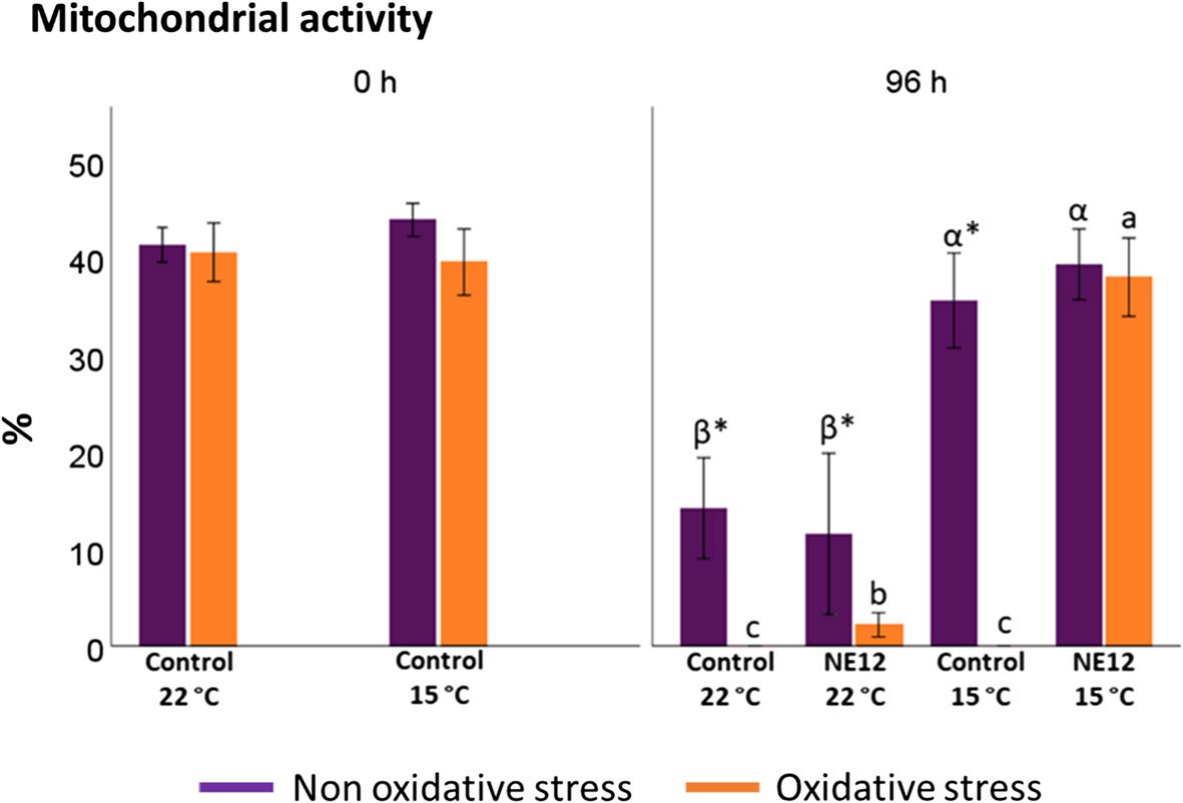
Effect of vitamin E nanoemulsion (NE) on mitochondrial activity: comparison between control and NE12 over time. Plots show the quadruple interaction of treatments × oxidative stress × incubation temperature × incubation time for the flow cytometry analysis of sperm viability. ^α,β^ (*P* < 0.05) indicate significant differences among treatments × temperature without oxidative stress at the same incubation time (purple line). ^a–c^ (*P* < 0.05) indicates significant differences among treatments × temperature under oxidative stress at the same incubation time (orange line). ^*^(*P* < 0.05) indicate differences between oxidative stress and non-oxidative stress treatments within each treatment. DNA Stainability, although the data is not shown in the figure

No significant differences in mitochondrial activity (ΔΨ_m_) were observed at any incubation time under both temperature conditions without oxidative stress (Fig. 6). However, under oxidative stress conditions, the best results after 96 h were achieved by NE12 at 15 ºC (control at 22 ºC: no mitochondrial activity; control at 15 ºC: 0.02 ± 0.01 vs. NE12 at 22 ºC: 2.19 ± 1.38; NE12 at 15 ºC: 38.01 ± 10.85) (Fig. 7). Throughout the entire incubation period, NE12 treatments consistently demonstrated better results than the control, particularly after 48 h.

### Effects of vitamin E nanoemulsion on ram sperm intracellular ROS production, lipid peroxidation and DNA

**Fig. 8:**
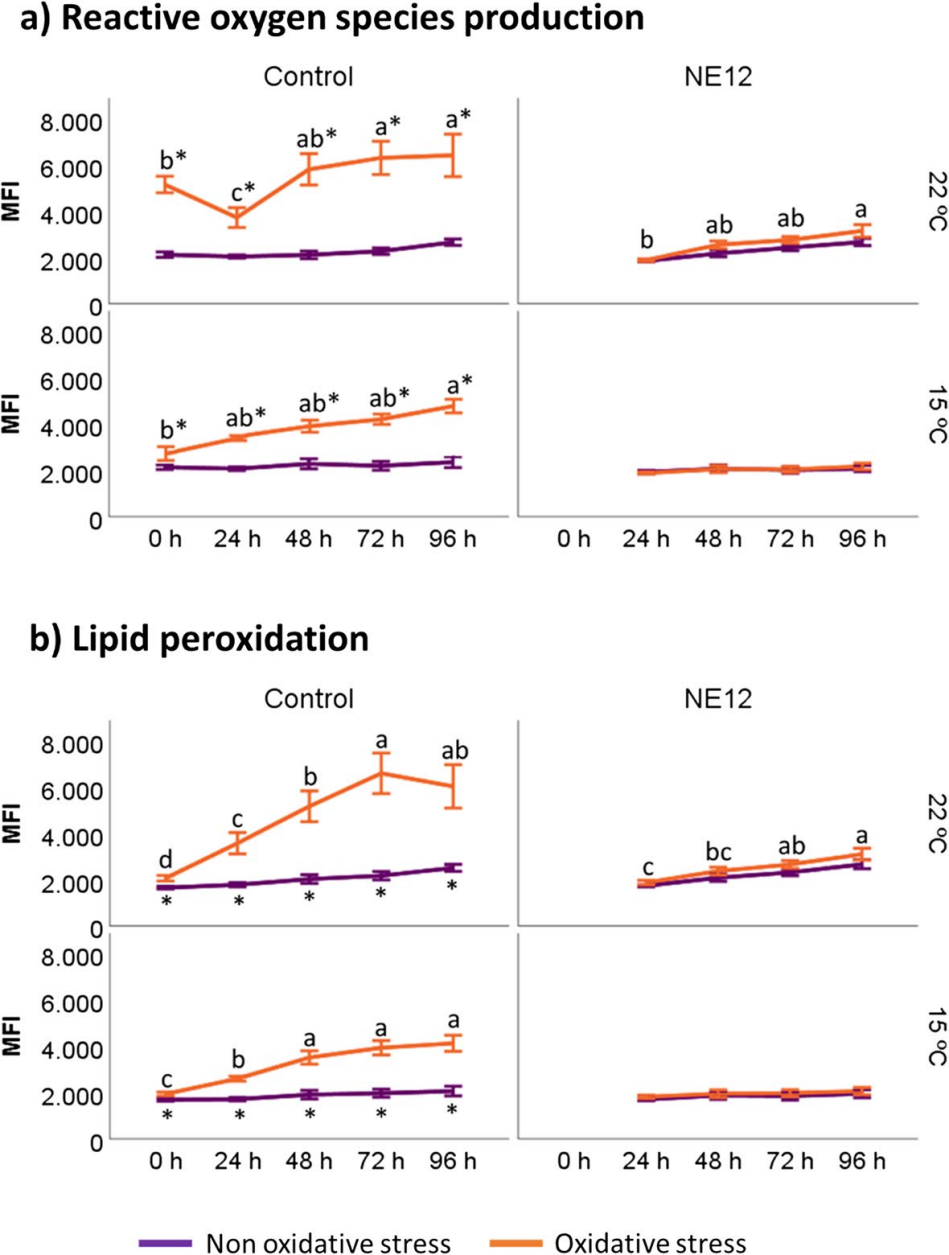
Effect of vitamin E nanoemulsion (NE) on: **a**) ROS production and **b**) lipid peroxidation. Plots show the quadruple interaction of treatments × oxidative stress × incubation temperature × incubation time for the flow cytometry analysis of reactive oxygen species (median fluorescence of H2DCFDA in PI− sperm) and lipid peroxidation (median green fluorescence of BODIPY C11). ^a–d^ (*P* < 0.05) indicates significant differences among hours of incubation within each treatment and temperature of incubation under oxidative stress (orange line). ^*^(*P* < 0.05) indicate differences between oxidative stress and non-oxidative stress treatments within each temperature and time of incubation

In general, the levels of lipid peroxidation were consistent with ROS production (Fig. 8). In the absence of oxidative stress, intracellular ROS did not exhibit significant differences between treatments at any time or temperature (Fig. 8a). However, under oxidative stress conditions and at both temperatures, significant differences were observed after NE12 treatment at all time points (*P* < 0.01). NE12 treatments effectively maintained ROS levels similar to those observed in non-oxidative stress treatments for up to 96 h (control 0 h non-oxidative stress at 15 ºC: 2136.80 ± 260.03 vs. NE12 96 h at 15 ºC: 2064.51 ± 418.83) (Fig. 8a).

**Fig. 9:**
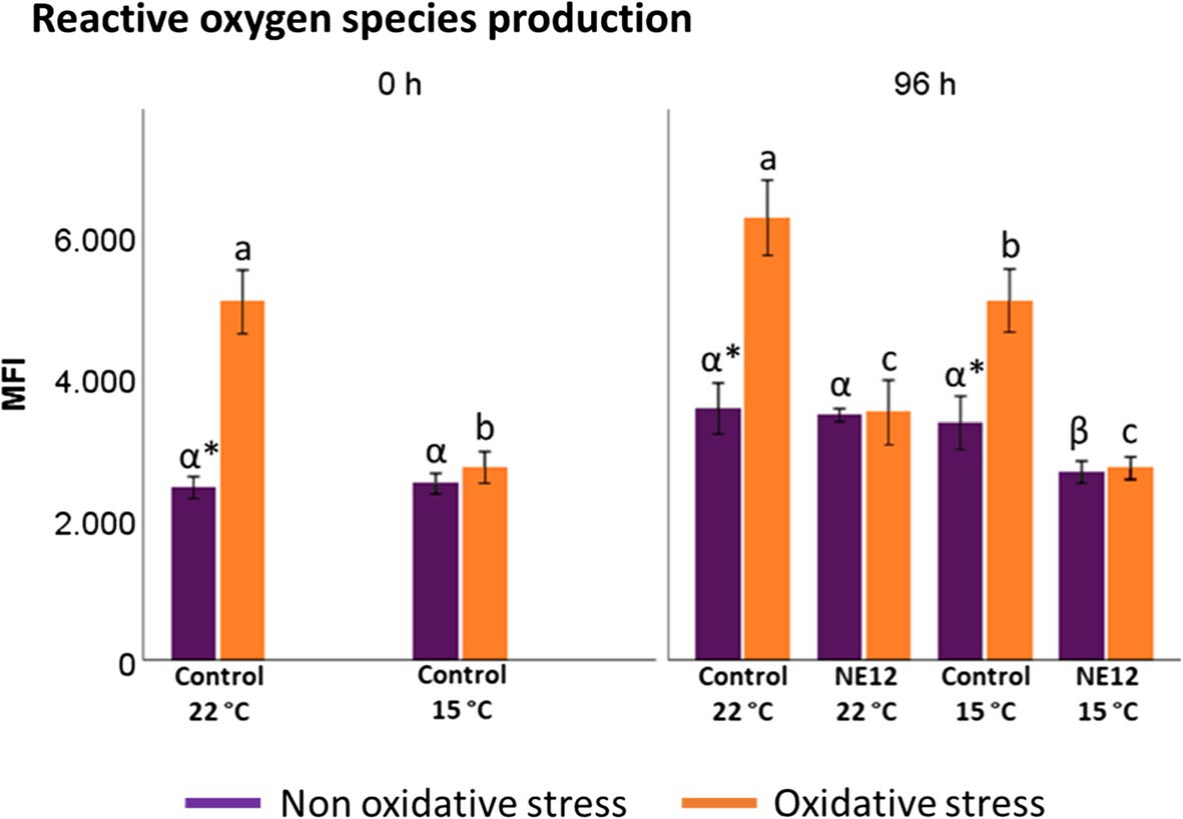
Effect of vitamin E nanoemulsion (NE) on ROS production: comparison between control and NE12 over time. Plots show the quadruple interaction of treatments × oxidative stress × incubation temperature × incubation time for the flow cytometry analysis of sperm viability. ^α,β^ (*P* < 0.05) indicate significant differences among treatments × temperature without oxidative stress at the same incubation time (purple line). ^a–c^(*P* < 0.05) indicates significant differences among treatments × temperature under oxidative stress at the same incubation time (orange line). ^*^(*P* < 0.05) indicate differences between oxidative stress and non-oxidative stress treatments within each treatment

Lipid peroxidation levels displayed a similar pattern to ROS production (Fig. 8b). NE12 treatments significantly reduced the BODIPY C11 fluorescence signal below the control in oxidized samples, maintaining levels close to those observed at 0 h of incubation at all time points and temperatures (Fig. 9).

**Fig. 10:**
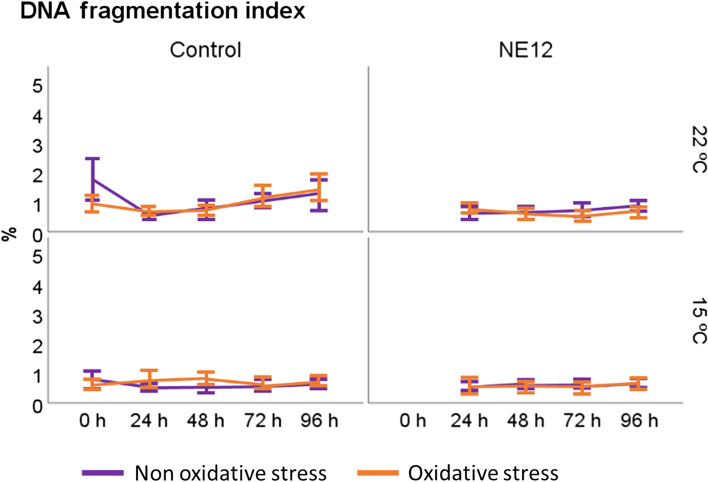
Effect of vitamin E nanoemulsion (NE) on DNA fragmentation. Plots show the quadruple interaction of treatments × oxidative treatment × temperature × incubation time for the flow cytometry analysis of sperm viability. No significant differences were observed

Figure 10 illustrates the results obtained for the DNA fragmentation index after 96 h of incubation under oxidative and non-oxidative stress conditions. No significant differences were observed between the treatments. Similar results were obtained for High DNA Stainability, although the data is not shown in the figure.

## Discussion

This work let us propose a better suitable media to improve quality in sperm storage for AI programs. The media might be of particular interest to those breeders working on ram species. Ram spermatozoa disclose a relatively short fertile lifespan which make them difficult to be transported, therefore making mandatory cryopreservation when considerable distances exist between livestock farms and reproduction centers [1, 7]. In this sense, NE previously described by some of us [30] has been studied as efficient tools for ram spermatozoa preservation at long intervals. The effects of NE were assessed on spermatozoa collected by artificial vagina and stored for 96 h at 22 or 15 ºC.

The relationship between temperature and sperm quality is crucial in both short-distance and long-distance semen transportation methods. In shorter distances, refrigeration at temperatures ranging from 0–15 °C is commonly employed to slow down the biochemical processes of spermatozoa. However, this cooling process can have negative effects on sperm fertility, with a reduction of 10%–35% per day [1]. Similarly, in long-distance transportation, cryopreservation is widely utilized, involving freezing the samples at −196 °C using liquid nitrogen [4]. Regardless of the method, cooling the spermatozoa leads to an increase in the percentage of immobile cells and abnormalities in their shape. Moreover, the distribution of membrane lipids is altered, and intracellular calcium levels rise, collectively referred to as cold shock [5]. These temperature-related changes significantly impact sperm quality and need to be carefully considered in order to preserve their reproductive potential during transportation.

To start with, the incorporation of NE into the media significantly improved sperm motility, following patterns previously reported in red deer (*Cervus elaphus hispanicus*) [12, 13] and ram (*Ovis aries*) spermatozoa [30]. However, in these previous reports, samples were incubated at 37 ºC and not for more than 24 h. In this study, we expand the study to more time and different temperatures to ascertain that NE maintain the quality of ram spermatozoa for up to 96 h without losing sperm motility. These results are particularly relevant because fertility is correlated with kinematic parameters in several species such as boar [31], bull [32], ram [33] or stallion [34]. The use of different commercially extenders has been tested in different species, such as alpaca [35] or stallion [36], looking forward to finding out a viable alternative to frozen semen which give rise to a loss of sperm motility. As far as we are concerned, this is the first study in animal spermatozoa in which the use the nanotechnology is applied to increase the viable lifespan for up to 96 h preserving all kinematic parameters. The beneficial effects of our lipid-based nanoparticles are due to their bioavailability, biocompatibility, biodegradability of major components, low toxicity, and the ability to carry hydrophobic compounds such as VE. The report by Soares et al. [37] is a good example on the difficulty of finding viable carriers for showing beneficial effect on post-thawing board sperm quality.

Then, the preservation of spermatozoa motility was positively correlated with the results on mitochondrial activity. Mitochondria is an organelle key in several process, such as ATP synthesis and sperm motility, and any damage to them could lead to apoptosis. In this case, sperm cryopreservation is a procedure where the mitochondrial membrane potential integrity is compromised [38]. Several studies reported that oxidative phosphorylation performed by mitochondria is the main source of ATP production required for flagellar movement and that mitochondrial alterations are related with loss of sperm motility [39]. In the current study, the controlled release of vitamin E through nanoemulsions before incubation at 15 and 22 ºC, increased mitochondrial potential in comparison to control group under exogenous oxidative stress conditions. Therefore, from the correlation between motility parameters and mitochondrial activity in samples supplemented with NE, we hypothesize that the protective effect of NE on sperm motility could be due to its effect on mitochondrial activity, together with the values obtained for acrosome integrity. These results are in accordance with our previous studies carried out on red deer [12, 13] and ram spermatozoa [30]. This relationship between spermatozoa motility and mitochondrial activity has been reported in other specie by other research groups. For example, Swegen et al. [40] proved a straight relationship between ATP content, mitochondrial activity, and sperm motility on stallion spermatozoa.

In addition, sperm viability is preserved for up to 96 h after NE treatments. These results open a new way in the use of AI, particularly in ram where the application of intracervical AI needs the use of liquid semen [41]. These samples must have a minimum shelf-life of between 2–4 d to be transported large distances [42]. In this study, the use of NE maintained the final percentage of live cells after 96 h near to control levels at 0 h. This effect was observed with and without oxidative stress conditions. At the same time and according with previous studies [12, 13, 30], a protective effect on acrosome integrity was observed. In accordance with the rest of the results, the NE showed a protective effect on the health of sperm DNA, which is an essential factor for fertilization [43]. All these results lead us to determine that the supplementation of medium extender with NE have a protective effect against cold shock on ram spermatozoa. This protective effect had been observed previously, but only with free vitamin E [44, 45].

Finally, the results showed that ROS production and lipid peroxidation levels decrease significantly in NE treatment with respect to control under oxidative stress conditions. This effect has been described previously in red deer [12, 14, 16, 21], bulls [46, 47], boars [48, 49] and humans [50] but never over such a long period of time. Through sperm storage, spermatozoa are subjected to several sources of oxidative stress, such as storage under low temperatures and the common oxidative metabolism in the mitochondrial [51]. This large amount of ROS is particularly critical on rams, where their spermatozoa show a high percentage of polyunsaturated fatty acids, which makes especially susceptible to lipid peroxidation in the presence of ROS [52]. At the same time, spermatozoa require a certain amount of ROS in order to control essential sperm functions, such as the acrosome reaction [53]. Therefore, the search of an appropriative balance between ROS scavenging and production is crucial. In this study, we ascertain that the use of NE provides an optimal oxidative stress defense for up to 96 h reaching normal levels for lipid peroxidation. This result is remarkable due to an increase of lipid peroxidation makes a plasma membrane dysfunction with the consequent loss of fertilizing potential [54].

## Conclusion

Existing protocols for sperm samples transport must be improved in AI to take part in breeding programs, especially for species with short fertile lifespan such as ram. From this work, we propose the use of nanotechnology as a tool to minimize oxidative stress and cold shock during sperm transport for longer intervals of time. Our results show that NE could be used to maintain ram spermatozoa during transport at 15 and 22 ºC for up to 96 h, with no appreciable loss of kinematic and physiological characteristics of freshly collected samples. Our results will be particularly relevant when the situation of increased oxidative stress is not due to mimicking in the laboratory but to situations where it actually increases due to exposure to toxins, prolonged sample transport, suboptimal sample storage, poor nutrition, or presence of infectious diseases. The storage of ram spermatozoa in liquid form for 2–5 d may lead more flexibility to breeders in AI programs. In view of the potential and high versatility of these nanodevices, further studies are being carried out to assess the proposed sperm preservation medium on fertility after artificial insemination.

## Data Availability

The data presented in this study are available on request from the corresponding author. The data are not publicly available due to privacy.

## Abbreviations

AI: Artificial insemination
ATP: Adenosine triphosphate
DFI: DNA Fragmentation Index
DLS: Dynamic light scattering
HDS: High DNA Stainability
MMP: Mitochondrial membrane potential
NE: vitamin E nanoemulsion
PDI: Polydispersity index
PI: Propidium Iodide
PM: Progressive motile sperm
PNA: Peanut agluttinin
R_H_: Hydrodynamic radio
ROS: Reactive oxygen species
SCA: Sperm class analyzer
TM: Total motile sperm
VAP: Average path velocity
VCL: Curvilinear velocity
VSL: Straight-line velocity path
